# Screen Time of Preschool-Aged Children and Their Mothers, and Children’s Language Development

**DOI:** 10.3390/children9101577

**Published:** 2022-10-18

**Authors:** Riikka Mustonen, Ritva Torppa, Suvi Stolt

**Affiliations:** Department of Psychology and Speech-Language Pathology, Faculty of Medicine, University of Helsinki, P.O. Box 21, 00014 Helsinki, Finland

**Keywords:** screen use, digital devices, co-view, lexical skills, language ability

## Abstract

Although children’s increased screen time has been found to associate with poorer language development, it is open to question which part of language ability screen time specifically associates with. Our aim was to examine the association between children’s screen time (alone and together with a parent), mothers’ screen time, and the different domains of children’s language skills. Mothers reported their children’s (N = 164, aged 2.5 to 4.1 years) screen time and their own on a weekday and a day off. Children’s lexical, phonological, morphological, receptive, and general language abilities were measured using validated tests. The connections between children’s and mothers’ screen time and children’s language skills were analyzed using correlation analyses and linear regression models. The more the children used screen time alone, or the greater the amount of the mothers’ screen time, the weaker the children’s lexical and general language abilities when the children’s age, maternal education level, and birth order were controlled for. We also found cumulative, negative links to the children’s lexical and general language abilities when the amount of their screen time alone and the amount of the mothers’ screen time were simultaneously included in the regression model. The results suggest that it is important to restrict both children’s screen time spent alone and mothers’ screen time.

## 1. Introduction

The definition of screen time includes time spent using multiple devices such as the TV, mobile devices, computers, and game consoles [1]. The American Academy of Pediatrics and the World Health Organization recommend no more than one hour of screen time for two- to four-year-old children, preferably educational content, viewed together with a parent [2,3]. However, the average screen time of children often exceeds recommendations. For example, the average amount of screen time of American children aged two to four was 150 min per day [1]. In a Finnish study, the average screen time of children aged three to six was 111 min per day [4]. Early childhood is a critical phase for language acquisition. During the early years, the different domains of language (i.e., lexicon—vocabulary; phonology—the ability to use phonemes based on the rules of one’s native language; morpho-syntax—the ability to use and comprehend inflections and sentence structures based on the rules of one’s native language; pragmatics—the ability to use language that is typical of the language context in question) are acquired through interaction with adults. The current concern is that screen time reduces the amount and quality of interaction between children and parents, leading to fewer opportunities for the child to practice their language skills [5,6].

Several studies have indeed found that children’s increased screen time is associated with poorer language development [7,8,9,10,11]. However, some studies have not detected such detrimental association between children’s screen time and language skills [12,13]. In addition, previous research suggest that co-viewed screen time with a parent may hinder the negative effects of screen time associated with language development, or even facilitate children’s language acquisition [7,14]. Thus, to date, the effect of screen time on language skills is unclear, possibly due to the differences in the variables used in different studies. For example, some studies have separated children’s screen time spent alone and the co-viewed screen time [15,16], while other studies have explored children’s total amount of screen time [8,12,13]. Above all, existing studies investigating the effect of screen time on children’s language development have only utilized a general language score or the results of brief screening tests as an index for language ability [10,14,17], or only focused on lexical skills [8,16,18]. Thus, although screen time might affect different language domains differently [19], previous studies have not answered the question of which language domains screen time use specifically influences.

Maternal sensitivity (i.e., the ability to respond promptly and appropriately to a child’s initiatives; [20]) promotes language development [20,21]. Sensitive mothers who respond verbally to their children’s initiatives provide more language input for their children than mothers who respond rarely [20,21,22]. Moreover, children need uninterrupted joint attention and meaningful and temporally contingent interactions to learn novel words [23]. Mothers’ screen usage might compromise responsive joint attention. Recent research supports an association between mothers’ active digital usage and reduced parent–child interaction [24,25,26]. Similarly, the screen time of children may reduce interaction opportunities [5,6]. However, according to our knowledge, no prior studies have investigated the possible effect of both children’s and mothers’ screen time together on the children’s development of language skills, although this is a relevant societal question in our modern world which actively utilizes different screen devices.

The research questions were as follows: (1) Is the amount of children’s screen time, alone and together with a parent, or that of the mothers associated with children’s expressive lexicon, phonology, morphology, receptive language ability, or general language level? (2) How much the screen time of children, the screen time of their mothers, or both at the same time explain the possible variation in children’s language skills when the effects of background factors (children’s age, maternal education level, and birth order) are controlled for? We hypothesized that a higher amount of children’s screen time will associate with weaker language skills since the opportunities for responsive language input are reduced due to active use of screen time [5,6], especially when the screen time is spent alone. Further, we hypothesized that a higher amount of mothers’ screen time will associate with children’s weaker language skills because the greater the mothers’ screen time, the less frequent the joint attention moments between a mother and a child [23,24,26].

## 2. Materials and Methods

### 2.1. Participants

The participating children and parents were recruited between spring 2019 and spring 2020 before the COVID-19 pandemic reached Finland. The inclusion criteria were normal development and Finnish as the native language. The exclusion criteria were cognitive delay, hearing impairment, developmental language disorder, and autism spectrum disorder. Information on the study was sent to the directors of randomly chosen daycare centers in the Helsinki district (capital area in Finland). The daycare centers’ workers distributed the study information to families that met the inclusion criteria. After signing their written consent, the families that were willing to participate received the research materials by mail, and a separate time was booked for the child’s language assessment. Language assessment was carried out by trained Speech-Language Pathology students at the daycare centers.

The study sample comprised 164 monolingual Finnish-speaking children aged 2.5 to 4.1 (mean age 3.4 years; 49% boys) and their mothers. Table 1 presents the participants’ background information. The daily amount of spoken Finnish was at least 74% among all the participating children (only Finnish used at home: *n* = 147, 90%; other language than Finnish also used at home: *n* = 9, 5%; language information missing: *n* = 8, 5%). The majority were full term (born at > 37 gestational weeks), healthy children, but eight children (5%) were born before the 37th week of pregnancy. As these children had no major neurological diagnoses (exclusion criteria of the present study), their data were included in the study. All education levels were represented in the maternal and paternal education backgrounds. The education level of the mothers was slightly higher than that of the Finnish population in general but reflected the educational structure of young Finnish adults in the Helsinki district, where the education level is slightly higher than that of the general Finnish population [27].

This cross-sectional study is part of the validation and norming study of the Finnish version of the MacArthur Communicative Development Inventories III (LEINIKKI Study; FinCDI III; principal investigator: last author of the present study). The University of Helsinki Ethical Review Board in Humanities and Social and Behavioral Sciences (Statement 2/2018) approved the protocol of the LEINIKKI Study. Parents signed their written informed consent before participating in the study and received written feedback on their children’s test results. If a child had any problems in their language development, they were referred to public health care.

### 2.2. Measures

The mothers completed The Screen Time Questionnaire (STQ [28]; see also [29]) for both themselves and their children. Screen time was defined as time spent watching TV and watching or using mobile devices, computers, laptops, and game consoles. The following six open-ended questions were asked (total time in hours and minutes): How much time does your child spend using screen devices on a weekday/day off? How much of this time is spent with a parent (co-viewing in hours and minutes) on a weekday/day off? How much time do you (the mother) spend using screen devices on a weekday/day off? The STQ has been used in Estonian children [28]. Comparable measures have been used in other studies as well [6,29].

Information on expressive lexical skills was gathered using the vocabulary section of the Finnish version of the MacArthur Communicative Development Inventories III (FinCDI III Words [30]; see also [31,32]). The FinCDI III has been adapted on the basis of the Swedish version of the CDI III [32]. The content of the vocabulary section of the FinCDI III corresponds closely to that of the Swedish version. The validation of the FinCDI III is ongoing [30]. The FinCDI III Words contains a checklist of 100 words from four thematic themes (food words, body words, mental words, and emotion words). The parent reports whether their child spontaneously uses the word on the list (max 100 points, Swedish median values: 51 points for 2.5-year-olds, 62 points for 3.0-year-olds, 72 points for 3.5-year-olds, and 80 points for 4.0-year-olds [32]).

The Finnish Phonology test (FPT [33]) and the Finnish Morphology test (FMT [34]) were used to test the components of language structures. Both are standardized tests validated for the Finnish population. The FPT measures a child’s phoneme inventory and their ability to combine phonemes according to the rules of the Finnish language [33]. During the test, the child names 36 or 90 pictures, depending on their age (children younger than three: 36 pictures, max 60 points; children older than three: 90 pictures, max 127 points). The raw points were converted into percentile values, which were used in the present study. According to the FPT manual, a percentile value below 17 is considered weak phonological development at all ages. The short version of the FMT, which was used in the present study, measures how a child can use five different inflectional Finnish morphemes (comparative, superlative, elative, present tense, and past tense; max 75 points) [34]. The child inflects unfamiliar words with the help of pictures. For the FMT raw points were used (mean values: 7.46 points for 2.5-year-old children, 16.96 points for 3.5-year-old children, and 31.52 points for 4.5-year-old children).

The receptive part of the Reynell Developmental Language Scales III (RDLS III [35]) and the total score of the Finnish version of the MacArthur Communicative Development Inventories III (FinCDI III Total [30]) were used to measure the children’s receptive and expressive general language ability. The RDLS III has been standardized and validated for Finnish children, and the receptive part measures receptive vocabulary, comprehension of spatial concepts, comprehension of short and complex sentences, and reasoning ability [35]. For the RDLS III, standard scores were used (mean value of the norming group: 100 standard points; ±1 SD = 15 standard points). The FinCDI III Total score provided information on expressive language ability at large. The total score of the FinCDI III contains six parts that measure the child’s general level of communication (max 6 points), expressive lexicon (FinCDI III Words; max 100 points), ability to inflect words and language structures (16 points), complexity of language (20 points), speech clarity (phonology; 7 points), and metalinguistic skills (7 points; total max 156 points) [30].

### 2.3. Statistical Analyses

The screen time of the children and the mothers on a weekday and on a day off were first transformed into daily averages using the formula: [5 × screen time on a weekday + 2 × screen time on a day off] ÷ 7. Pearson’s correlations and partial correlations (controlled for age) were used to examine the associations between the amount of daily screen time of the children (alone and with a parent) and their mothers and the children’s language skills.

Altogether, eight linear regression models were used to assess the explanatory value of screen time for lexical or general language ability. In four models, the dependent variable was lexical ability, measured using the vocabulary section of the FinCDI III; and in four models, the dependent variable was general language ability, measured using the total score of the FinCDI III. The following background factors were entered into all the models: children’s age, maternal education level and birth order. Two models were run for each of the following explanatory variables: screen time of the children alone, screen time of the children with a parent (co-view), and screen time of the mothers. In addition, to analyze the possible cumulative effect of the children’s and mothers’ screen time, both the screen time of the children alone and the screen time of the mothers were included in the last two models. In all eight models, in the first step of the analysis, we ran a model of background factors for both dependent variables. In the second step, explanatory screen time variables were added to the models separately, which allowed us to test the added value of each screen time variable for the models (R^2^ change). No multicollinearity of the explanatory factors was detected in any of the models. Based on the preliminary correlational analyses between background factors and screen time and language variables (Appendix A), the chosen background factors were considered as possible confounding variables. The children’s age, maternal education level, and birth order associated significantly with both screen time and the language variables (Appendix A) and were therefore included in the regression models. Earlier studies have also reported comparable associations [36,37,38,39,40,41].

The percentage of missing values across the variables varied from 0 to 9.8%. All the available data were included in the analyses, and no imputations were made to the dataset. IBM SPSS statistics, version 26 for Windows was used to analyze the data. The level of significance was 0.05 in all the analyses.

## 3. Results

### 3.1. Data Description

The average daily screen time of the children was 79 min, of which screen time alone was 44 min and screen time spent with a parent 34 min (Table 2). The average daily screen time of the mothers, including during working hours, was 5 h 34 min (Table 2).

The expressive lexical skills of the children represented roughly typical performance in comparison to the Swedish norms (median of raw points of the present study: 59 points for 2.5- to 2.9-year-old children, 70 points for 3.0- to 3.49-year-old children, and 74 points for 3.5- to 4.1-year-old children). In terms of language structure, most of the children performed in line with their age level (median of the percentile values of phonological skills: 64; mean value of raw points of morphological skills: 7.29 points for 2.5- to 2.9-year-old children, 25.9 points for 3- to 3.9-year-old children and 29.82 points for 4- to 4.1-year-old children). However, seven children (4%) had weak phonological skills (<17 percentile value). Based on RDLS III, the receptive language skills of 122 children (74%) were typical (mean value of standard points: 103.97 points; Table 2). Twelve (7%) children had weak (<1 SD) receptive language skills. Variation in general language ability (FinCDI III Total) was high among individual children (Table 2). In the present sample, roughly 70% of the children (n = 119) scored between 75 and 135 points. Sixteen children (lowest 10%) had the weakest scores (<75 points).

### 3.2. Associations between Amount of Screen Time and Children’s Language Skills

Table 3 shows the results of the bivariate and partial correlations. The greater amount of screen time the children had alone, the poorer their general language ability (FinCDI III Total) when their age was controlled for (Table 3). Moreover, the more the children’s daily screen time was spent with a parent (co-view), the better their expressive lexical skills (FinCDI III Words), phonological skills (FPT), and general language ability (FinCDI III Total; Table 3). Furthermore, higher amounts of mothers’ screen time were significantly associated with weaker expressive lexical skills (FinCDI III Words) and general language ability (FinCDI III Total) among the children (Table 3).

### 3.3. Explanatory Value of Screen Time for Children’s Lexical and General Language Skills

**Figure 1:**
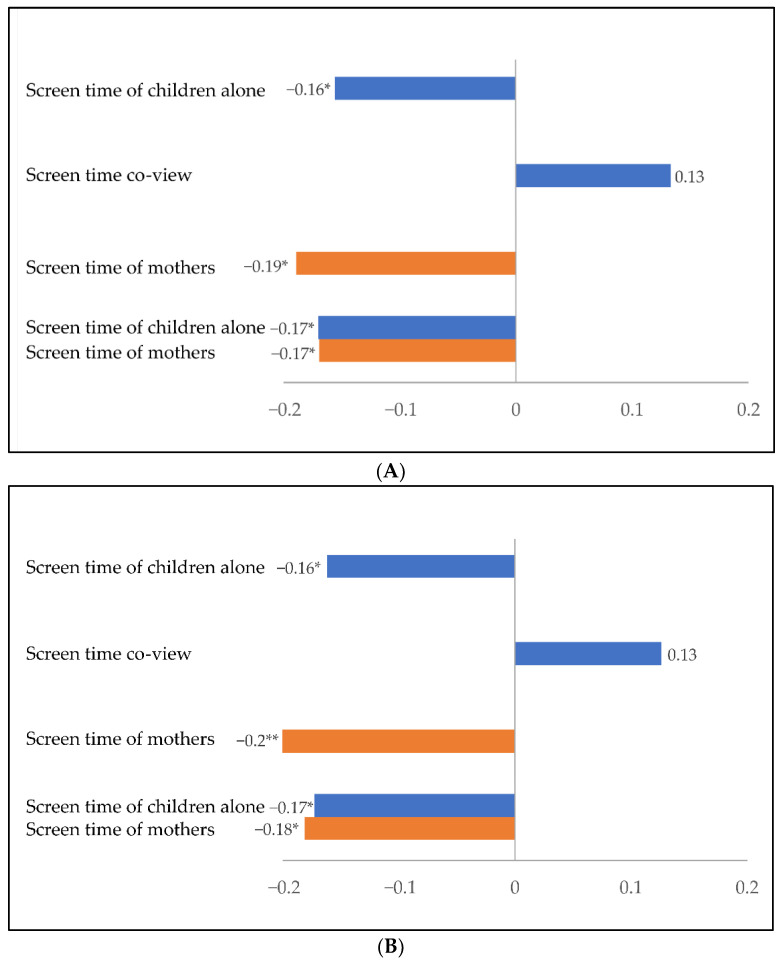
Standardized Beta-values of the screen time variables from the regression models for the dependent variables FinCDI III Words (score of vocabulary section of Finnish version of MacArthur Communicative Development Inventories III; (**A**) and FinCDI III Total score (**B**). A negative value indicates a negative association between the screen time variable and the language skill. The blue bars represent children’s screen time, and the orange bars represent mothers’ screen time. The last two Beta-values at the bottom of the charts represent the results from the combined models (including both the screen time of children alone and the screen time of mothers). Children’s age, maternal education level, and birth order were controlled for in all models. * *p* < 0.05; ** *p* < 0.01.

All the regression models were significant (Table 4, Figure 1). The first two models with expressive lexical and general language ability as a dependent variable and the screen time of children alone as an explanatory variable together with the background factors explained 28% of the variation in expressive lexical ability and 30% of the variation in general language ability. In both models, the change of the R-square was significant after adding the children’s screen time alone to the models. As indicated by the negative beta-value, the more screen time the children had alone, the poorer was their lexical and general language ability when background factors were controlled for (Table 4, Figure 1). As the amount of screen time alone increased by, for example, 30 min, expressive lexical skills decreased by 2.5 points (minutes × B of FinCDI III Words = 30 × −0.084). Similarly, as the amount of screen time alone increased by 30 min, general language skills decreased by 4.0 points (minutes × B of FinCDI III Total = 30 × −0.133).

The next two models, which included the amount of screen time with a parent (co-view) and the background factors as explanatory variables, explained 27% of the variation in expressive lexical ability and 29% of the variation in general language ability (Table 4). In both models, screen time with a parent had positive beta-values (Figure 1), indicating that the more screen time that was spent with a parent, the higher was the children’s expressive lexical and general language abilities. However, the screen time spent with a parent did not reach the significance level of 0.05 (lexical skills: t = 1.84, *p* = 0.07, after bootstrapping *p* = 0.052; general language skills: t = 1.74, *p* = 0.08, after bootstrapping *p* = 0.06) in either model when background factors were controlled for (Table 4).

The next two models, with the amount of screen time used by the mothers and the background factors as explanatory variables, explained 29% of the variation in expressive lexical ability and 32% of the variation in general language ability (Table 4). The screen time of the mothers was a significant explanatory variable (Figure 1), and the R-square change was significant when the mothers’ screen time was added to both models. The more screen time the mothers had, the poorer were the expressive lexical and general language abilities of their children. As the mothers’ screen time increased by 120 min, the children’s expressive lexical skills decreased by 2.8 points (minutes × B of FinCDI III Words = 120 × −0.023). In addition, as the mothers’ screen time increased by 120 min, the children’s general language skills decreased by 4.4 points (minutes × B of FinCDI III Total = 120 × −0.037).

The last two models (Table 4, Figure 1) included the amount of children’s screen time alone and the amount of mothers’ screen time as explanatory variables together with background factors. The last models had the highest explanatory value for the expressive lexical (31%) and general language ability (34%) of all the models. Both the children’s screen time alone and the mothers’ screen time were significant explanatory factors, as was the R-square change in both models. Thus, the more screen time the children and the mothers had in total, the poorer were the expressive lexical and general language abilities of the children.

## 4. Discussion

This study provided detailed information on the association between children’s and mothers’ screen time and children’s language skills. The correlation analyses showed a significant, although moderate, negative association between the screen time of children alone and their general language ability. In addition, based on the regression models, our results showed that the more screen time the children had alone, the poorer were their expressive lexical and general language abilities when their age, maternal education level, and firstborn child status were controlled for. In contrast, we found a positive association between the children’s screen time together with a parent (co-view) and the children’s expressive lexical, phonological, and general language abilities. However, after controlling for the background factors in the regression models, the screen time together with a parent did not remain a significant predictor of expressive lexical and general language abilities. Regarding the screen time of mothers, the results of the correlation and regression analyses revealed that the more screen time they had, the poorer were the expressive lexical and general language abilities of the participating children. Overall, the regression models showed that children’s screen time alone or mothers’ screen time, together with the background factors, explained 28% to 32% of the variation in lexical skills and general language ability. Most importantly, the regression models explained the most variation (31% to 34%) in the children’s expressive lexical and general language abilities when they included both the children’s screen time alone and the mothers’ screen time.

### 4.1. Screen Time of Children and Mothers

The results showed that poorer expressive lexical skills and poorer general language ability were related to a higher amount of children’s screen time spent alone. Previous studies have reported parallel associations between the screen time of children and their expressive vocabulary (lexical skills) and expressive language in general [8,16,19]. However, the present study analyzed the effect of screen time on children’s language skills in detail, which enabled us to verify that screen time may specifically influence children’s lexical and general language ability development. This result may be explained by the fact that the most common reason that children under eight years of age use screen devices is still to watch TV or videos [1], which is passive in nature. Thus, because screen time is often passive and reduces child vocalizations [5], a child does not practice expressive lexical and expressive language skills during screen time by, for example, repeating words or phrases, or discussing the meaning of a word with an adult, especially if screen time is spent alone. In addition, findings of studies that have examined novel word learning through videos, television, or live chat have suggested that children need live social interaction in order to learn vocabulary [23,42,43,44].

However, opposite results regarding the associations between children’s screen time and language skills have also been found [12,13]. For example, a study that focused on highly educated families living in the UK found no significant associations between television viewing and mobile device use and size of vocabulary among 6- to 36-month-old children [13]. In another study, from preschool to the third grade, children participated in assessments at two time points, in the fall and in the spring of the academic year [12]. The parents reported the typical amount of media use on a school day. No significant association was found between media use and receptive and expressive vocabulary when the results of the assessments from both time points were included in the model. Methodological differences in study designs may explain the differences between the present and previously opposite results. Neither of these studies with opposite results specified whether the screen time was spent alone or with a parent, factors which may both affect language skills differently and thus may mix results.

In fact, we found a positive association between the children’s screen time together with a parent (co-view) and their expressive lexical, phonological, and general language abilities. However, in the regression analyses of lexical and general language abilities, the explanatory value of the children’s screen time together with a parent no longer remained significant when the effect of the children’s age, maternal education level, and firstborn status were controlled for. This finding is contrary to those of a previous study which suggested that co-viewed screen time may have a positive effect on lexical development [15]. These mixed results might be related to the fact that, although co-viewing offers an opportunity for interaction, the parent–child interaction during co-viewing may still be of a lower quality than the parent–child interaction in a non-digital context [45]. Carr and Dempster [45] compared parent–child mutual engagement in traditional toy conditions to digital tablet conditions and found that the interactions were more cooperative and warmer in the traditional toy conditions. Thus, for a child to learn language and have a rich language environment during co-viewed screen time, parents need to focus on interacting sensitively with the child and actively verbalizing, scaffolding, and discussing the content on the screen [46]. Otherwise, co-viewed screen time is as passive and non-interactional as screen time spent alone.

Another important finding was that the more screen time the mothers had, the poorer were the expressive lexical and general language abilities of their children. A recent study found that a smaller expressive vocabulary among 24-month-old children was associated with more television- and video-watching among parents [47]. Whether it is the children’s screen time or the mothers’ screen time, time spent in front of screens can mean fewer opportunities for children to interact with an adult and thus hinder language learning [5,25]. The screen time of mothers may not only reduce parent–child interaction; it may also interrupt and stop ongoing interaction [24,26]. Reed et al. [23] studied whether unpredictable interruptions affect word learning and found that children did not learn words if teaching was disrupted. Thus, as mobile smart devices are today common and always accessible in most households, technological interference with interaction is something to which parents should pay attention.

The regression models showed that a considerable portion of children’s expressive lexical skills and general language ability could be explained by the screen time of children or mothers together with the background factors. The results indicated a cumulative, negative effect on language development if both the child and the mother had a great amount of screen time. This is an important result and underlines the fact that screen time in families may indeed have an influence on children’s language skills. A recent study found that a higher amount of parents’ device usage was connected to a higher amount of screen time among children [24]. It is possible that children acquire their screen time behavior from their parents. However, to our knowledge, no previous studies have explored the cumulative effect of the screen time of both children and parents on children’s language development. Thus, the present finding concerning the cumulative effect of screen time is novel. Still, more research is needed to further explore this association.

### 4.2. Strengths and Limitations

The following issues are the strengths of the current study. The language skills of the children were evaluated thoroughly, which allowed us to expand on prior research by examining which language domains were associated with the screen time of children and mothers. Moreover, the present study included the screen time of mothers, and thus, for the first time, we were able to study the cumulative effect of the screen time of both children and mothers on the children’s language skills. However, the present study also had limitations. First, as in several other previous studies [6,29,48,49], we also used the parent report method to collect information on the amount of screen time, which might have caused under- or overreporting. However, our findings regarding the connection between screen time and language ability on a general level are consistent with those of previous studies. Second, our sample represented families living in the Helsinki district (capital area) in Finland; thus, the parents were more highly educated than Finnish parents in general. Therefore, although the findings provide representative information on screen time and the language skills of children living in well-educated families in the capital area, the education level of the families should be borne in mind when generalizing the results. Third, the content of screen time was not investigated, although the content as well may influence the development of language skills. Future studies should examine the connection between screen time content and children’s language skills carefully.

### 4.3. Clinical Implications

The present study is relevant for practitioners following the development of children and guiding families. The results justify recommending that parents should restrict the screen time that preschool-aged children spend alone. In addition, child’s screen time should be spent together with a parent. This recommendation is in line with that of the American Academy of Pediatrics of restricting the screen time of two- to four-year-old children to one hour and that this should be with a parent and should be educational in content [2]. Moreover, the negative association that the study found between the screen time of mothers and the language skills of children (lexical and general language ability) indicates that mothers should also limit their own screen time. The results of the cumulative negative effect of children’s and mothers’ total screen time on expressive lexical skills and general language ability suggest that the risk of a child having poor language abilities may grow if both the child and the mother have a great deal of screen time. Our findings propose that clinicians recommend restricting the screen time of both children and their mothers.

## 5. Conclusions

Our study provided detailed, comprehensive information on the association between screen time use and preschool-aged children’s language skills. The findings showed that a higher amount of children’s screen time alone and a higher amount of their mothers’ screen time is particularly associated with weaker lexical skills and weaker development of children’s general language abilities. The social context in which children learn language shapes their language development [22], which in turn is linked to later academic achievements, for example [50,51]. Thus, speech and language pathologists should include questions of family’s screen time as a part of the assessment of the child’s language ability. The present findings imply that future studies should examine the screen time of the whole family and explore in detail the possible cumulative effect of children’s and parents’ total screen time on language skills in general and lexical skills in particular. Longitudinal designs would also provide more understanding of how permanent the effects of children’s and parents’ screen time are on language development. In addition, since the data of this study was collected before the COVID-19 pandemic, future studies should investigate the effect of COVID-19 on the screen time used by children and their parents.

## Figures and Tables

**Table 1:** Background characteristics of participants (N = 164).

Characteristics	n (%)
Child	
Boy	81 (49)
First born	94 (57)
Day care	
Full-time Part-time	130 (79) 29 (18)
Parent	
Education of mothers	
Comprehensive school High school Bachelor’s degree Master’s degree or higher	3 (2) 17 (10) 27 (17) 112 (68)
Education of fathers	
Comprehensive school High school Bachelor’s degree Master’s degree or higher	4 (2) 36 (22) 33 (20) 82 (50)

Missing values: child’s gender n = 4 (2%), first born n = 8 (5%), day care n = 5 (3%), education of mothers n = 5 (3%), education of fathers n = 9 (6%).

**Table 2:** Descriptive statistics for screen time of children (alone and co-view) and mothers presented in minutes, and for children’s language test results.

Variable	M	SD	Min-Max
Screen time of children			
Weekday alone	40.34	30.16	0–150
Day off alone	54.72	40.34	0–210
Daily average alone	44.45	31.22	0–167
Weekday co-view	30.53	28.19	0–120
Day off co-view	43.92	37.58	0–210
Daily average co-view	34.35	29.39	0–135
Screen time of mothers			
Weekday	397.17	186.20	30–720
Day off	178.81	80.39	15–360
Daily average	333.54	138.35	26–579
Language skills of children			
FinCDI III Words	67.8	16.77	7–100
FPT (percent.)	60.3	25.31	4–98
FMT	23.18	14.60	0–62
RDLS III Receptive (sp)	103.97	12.51	50–127
FinCDI III Total	105.29	25.66	14–151

Co-view = children’s screen time with a parent; FinCDI III Words = score of vocabulary section of Finnish version of MacArthur Communicative Development Inventories III; FPT (percent) = Finnish Phonology Test, percentile values; FMT = Finnish Morphology Test; RDLS III Receptive (sp) = Receptive part of the Reynell Developmental Language Scales III, standard points. The number of participants varied between 148 and 159 in different measures.

**Table 3:** Pearson’s correlation co-efficient values (r) between screen time of children and mothers and children’s language ability. Partial correlations (rp; children’s age controlled) are shown separately.

	Lexicon	Structure		General Language Ability	
Screen Time (Daily Average)	FinCDI III Words	FPT	FMT	RDSL III Receptive	FinCDI III Total
Children alone r	−0.10	−0.12	−0.03	−0.10	−0.12
Children alone rp	−0.16	−0.13	−0.08	−0.10	−0.18 *
Children co-view r	0.22 **	0.19 *	0.003	0.07	0.22 **
Children co-view rp	0.19 *	0.17 *	−0.07	0.07	0.19 *
Mothers r	−0.29 **	−0.09	−0.10	−0.05	−0.30 **
Mothers rp	−0.24 **	−0.06	−0.01	−0.05	−0.25 **

* *p* < 0.05, ** *p* < 0.01; Children co-view = children’s screen time with a parent; FinCDI III Words = score of vocabulary section of Finnish version of MacArthur Communicative Development Inventories III; FPT = Finnish Phonology Test; FMT = Finnish Morphology Test; RDLS III Receptive = Receptive part of the Reynell Developmental Language Scales III. The number of participants varied between 137 and 154 in the Pearson’s correlation analyses, and between 135 and 152 in the partial correlation analyses.

**Table 4:** Results of linear regression analyses.

	FinCDI III Words					FinCDI III Total				
Explanatory Variables	B	*p* B	R^2^adj	R^2^ch	*p* Model	B	*p* B	R^2^adj	R^2^ch	*p* Model
Screen time of children alone	−0.08	0.03 *	0.28	0.02 *	<0.001 **	−0.13	0.02 *	0.30	0.03 *	<0.001 **
Screen time co-view	0.08	0.07	0.27	0.016	<0.001 **	0.11	0.08	0.29	0.014	<0.001 **
Screen time of mothers	−0.02	0.01 *	0.29	0.03 *	<0.001 **	−0.04	0.007 **	0.32	0.04 *	<0.001 **
Screen time of children alone	−0.09	0.02 *	0.31	0.06 **	<0.001 **	−0.14	0.02 *	0.34	0.06 **	<0.001 **
and screen time of mothers	−0.02	0.02 *				−0.03	0.01 *			

* *p* < 0.05; ** *p* < 0.01; screen time co-view = children’s screen time with a parent; FinCDI III Words = score of vocabulary section of Finnish version of MacArthur Communicative Development Inventories III; B = unstandardized beta coefficient, shows direction of connection; *p* B = significance of the B; R^2^adj = adjusted R-square of the model including background factors (children’s age, maternal education level and firstborn child status); R^2^ch = change of the R-square after adding screen time variable; *p* Model = significance of the regression model. The number of participants varied between 144 and 154 in different models.

## Data Availability

The data of the present study is not openly available, as the participants in this study did not agree to their data being shared publicly during the data collection phase of the study.

## Appendix A

**Table A1:** Pearson’s correlation co-efficient values (r) between background variables and screen time of children and mothers, and children’s language abilities.

	Screen Time			Language Skills				
Background Variables	Child Alone	Child Co-View	Mother	FinCDI III Words	FPT	FMT	RDSL III Receptive	FinCDI III Total
Maternal education level	−0.15	−0.16 *	0.11	−0.17 *	0.03	0.01	0.09	−0.13
Children’s age	0.08	0.11	−0.17 *	0.49 **	0.18 *	0.54 **	0.00	0.52 **
Birth order	0.13	−0.26 **	0.31 **	−0.15	−0.19 *	0.05	−0.11	−0.18 *
Gender	−0.03	0.02	0.10	−0.20 *	0.01	−0.15	−0.16 *	−0.21 *
Preterm birth	0.05	−0.06	−0.02	−0.23 **	−0.17 *	0.11	−0.23 **	−0.23 **

* *p* < 0.05, ** *p* < 0.01; Birth order (0 = firstborn, 1 = older siblings); Gender (0 = girl, 1 = boy); Preterm birth (0 = no, 1 = yes); FinCDI III Words = score of vocabulary section of Finnish version of MacArthur Communicative Development Inventories III; FPT = Finnish Phonology Test; FMT = Finnish Morphology Test; RDLS III Receptive = Receptive part of the Reynell Developmental Language Scales III; Children co-view = children’s screen time with a parent; The number of participants varied between 143 and 158.
